# Effectiveness of resilience-based interventions in schools for adolescents: a systematic review and meta-analysis

**DOI:** 10.3389/fpsyg.2023.1211113

**Published:** 2023-10-06

**Authors:** Maria Llistosella, Blanca Goni-Fuste, Leandra Martín-Delgado, Andrea Miranda-Mendizabal, Berta Franch Martinez, Carmen Pérez-Ventana, Pere Castellvi

**Affiliations:** ^1^Primary Health Care, Consorci Sanitari de Terrasa, Terrassa, Spain; ^2^Department of Nursing, Universitat International de Catalunya, Sant Cugat del Vallés, Spain; ^3^Teaching, Research and Innovation Unit, Fundació Sant Joan de Déu, Sant Boi de Llobregat, Spain; ^4^Mental Health Networking Biomedical Research Centre (CIBERSAM), Madrid, Spain; ^5^Department of Medicine, Universitat International de Catalunya, Sant Cugat del Vallés, Spain

**Keywords:** resilience, adolescence, school intervention program, systematic review, meta-analysis

## Abstract

**Introduction:**

Resilience has been identified as a dynamic process that provides capabilities to face adversity. Considering the many protective factors involved in resilience and that the school is a key context to promote resilience, this review aimed to examine the effect of school-based interventions on resilience in adolescents.

**Methods:**

A systematic literature review and meta-analysis were conducted in July 2021 on four databases. The risk of bias was assessed using the Cochrane risk of bias tool. Random-effects meta-analysis was used to obtain pooled estimates. Stratified analyses were done according to population type (general, at risk), intervention type, and follow-up assessments.

**Results:**

Of the 1,667 articles obtained, 27 were included in the systematic review and 16 in the meta-analysis. The random effects indicated a significant increase in resilience after the intervention [SMD = 0.58, 95% CI (0.29–0.87)]. Subgroup analysis showed effectiveness only in the population at risk [SMD = 1.28, 95% CI (0.53–2.03)] and early adolescence [SMD = 1.28, 95% CI (0.42–2.14), PI (−7.44 to 10.33)]. Multicomponent intervention [SMD = 1.45, 95% CI (0.11–2.80)] and Cognitive Behavioural Therapy (CBT) [SMD = 0.20, 95% CI (0.06–0.34)] demonstrated substantial effectiveness. Significant results were observed within 8-week follow-ups or less [SMD = 1.55, 95% CI (0.61–2.48)].

**Discussion:**

These findings provide evidence that multicomponent and CBT interventions increase resilience in early at-risk adolescents only in the short term. Developing resilience interventions is useful in schools exposed to unfavourable socioeconomic contexts. Furthermore, long-term interventions should be redesigned to improve their effectiveness.

**Systematic review registration:**

PROSPERO [CRD42021277493].

## Introduction

Poverty or low socioeconomic status, maltreatment and sexual abuse, poor quality family environment, negative life events, and parents with mental disorders, among others, can negatively affect the physical, mental, and social health of adolescents. The age of onset of most mental disorders is between 12 to 25 years-old, with 20% of the affected population being adolescents (Kessler et al., 2005; Wei et al., 2013). The incidence of mental disorders in adolescents has drastically increased over the past few years (World Health Organization, 2021). Fortunately, not all adolescents exposed to adversity and risk factors develop psychological distress or mental disorders; healthy adolescents, despite being at risk or exposed to adversity, may be defined as resilient.

Although there are several definitions of resilience, there is no clear or universally accepted one (Aburn et al., 2016). Connor and Davidson (2003) defined resilience as a psychological trait or quality that characterised people with a greater capacity to cope with adversity. Resilience is also identified as a dynamic process (Masten et al., 1999; Luthar and Cicchetti, 2000) in which resilient behaviours result from a positive adaptation to a risky environment (Masten and Obradovic, 2006). Defining resilience as a dynamic process implies that there is an association with individual qualities or traits, the risk context, and social and psychological outcomes (Masten et al., 2008; Supkoff et al., 2012). Many protective factors are involved in the resilience process. According to the recent Individual and Environmental Resilience Model (IERM), these protective factors are significantly associated with a lower incidence of mental disorders or other diseases (Llistosella et al., 2022). The IERM describes two major dimensions of resilience: the environmental—family, school, peers, and cultural and community domains and the individual—biological behaviour, communication, and cognitive and emotional domains. The main protective factors involved in the resilience process are spirituality, relationships and social support, family support, physical activity, coping and perseverance, self-efficacy, competence, self-regulation, empathy, self-esteem, and social skills (Llistosella et al., 2022).

Given the complexity of resilience and the high number of protective factors involved in the resilience process, several training programmes with various formats and durations have been carried out to improve resilience among different populations (Chmitorz et al., 2018). Interventions using Cognitive Behavioural Therapy (CBT) in combination with other strategies, such as mindfulness, have shown a positive impact on the general population, on individual resilience (Joyce et al., 2018), or on mental health, specifically reducing depression and anxiety symptoms (Dray et al., 2017). Few studies have also reported differences in the outcomes depending on the population's age (Pinto et al., 2021). Resilience-based interventions implemented at high schools have also been reported to improve resilience among adolescents (Pinto et al., 2021).

However, for resilience interventions that are planned at schools, there is a need (a) to better understand potential differences in the effectiveness of the interventions due to the characteristics of the adolescents, that is, if they are or have been exposed to any risk factor; (b) to extend the knowledge about the protective factors involved in resilient processes; and (c) to identify components or techniques that may be more effective and if the follow-up time may affect the effectiveness of the interventions.

Adolescence is the period of transition from childhood to adulthood, typically ranging between ages 10 and 19. However, it could also extend up to the age of 21. Accordingly, adolescence occurs in three stages: early (10–13 years old), middle (14–17 years old), and late (18–21 years old). Since it is also a sensitive period for the development of mental disorders, interventions for supporting adolescents with coping skills to deal with stressful life events have been encouraged (Dadaczynski et al., 2020). Schools are, therefore, one of the key environments for fostering resilience among this population (Greenberg, 2006). The purpose of this study is to enhance our understanding of resilience interventions by identifying key protective factors involved in the resilient process and determining the characteristics and components needed to increase the effectiveness and sustainability of existing and future interventions.

The questions that guided this systematic review were as follows: (a) How effective are resilience-based interventions for adolescents in schools (aged 10–19) compared to other wellbeing interventions or non-interventions? (b) Are there any differences in the effectiveness of resilience-based interventions for not-at-risk and at-risk adolescents? (c) Are there differences in the effectiveness of resilience-based interventions according to the follow-up period? and (d) Which components of the interventions are associated with increasing resilience? Effectiveness was considered as the intervention's performance under “real-world” conditions (Revicki and Frank, 1999).

## Objective

This review aimed to examine the effectiveness of resilience-based school interventions for the adolescent population and their effect size according to the target population, the type of intervention, and the duration of the intervention compared to other wellbeing interventions or non-interventions to increase resilience. In addition, we aimed to identify specific components in the interventions that may be associated with resilience.

## Methods

### Design

A systematic review of the literature and meta-analysis was conducted (Furlan et al., 2009) according to the Preferred Reporting Items for Systematic Reviews and Meta-Analyses (PRISMA) guidelines (Moher et al., 2009). Please see Supplementary material 1 for the PRISMA checklist. The protocol for this systematic review and meta-analysis was registered in PROSPERO [CRD42021277493].

### Eligibility criteria

#### Types of studies and interventions

The following original studies were included: studies assessing resilience group-based interventions in schools, which were non-randomised and randomised controlled trials (RCTs) and cluster trials (cRCTs), quasi-experimental studies, and studies using mixed methods. Only studies published in English and Spanish were included.

Eligible CBT interventions included those directly targeting resilience via a predominantly CBT-based psychological treatment. CBT interventions may enhance an individual's ability to deal with intrusive thoughts that may follow exposure to a potentially traumatic event in a context of vulnerability, risk, violence, or trauma. Interventions that were not predominantly CBT, such as mindfulness-based interventions and acceptance and commitment therapy, were not included in this definition. Furthermore, multicomponent interventions that were composed of more than one psychosocial intervention, such as problem-solving combined with mindfulness or social and emotional learning, were included.

Studies with results of interventions focused on resilience outside the school setting were excluded. Similarly, articles with no access to full text or raw data were not included. Conference proceedings, guidelines, dissertations, commentaries, letters, protocols, and pilot studies were also excluded. Previous systematic reviews were manually searched to locate eligible studies. The included studies were recorded as additional studies from other sources.

#### Type of participants

The eligibility criteria included adolescents (aged 10–19 years) from the general population and within a context of risk, violence, or trauma. The risk context was defined as exposure to traumatic experiences; being a victim of interpersonal violence; poverty; family problems; substance abuse, mental problems, or criminal behaviour by parents; war; natural disasters; pandemics; and being an immigrant (Llistosella et al., 2022). Studies that reported data from participants who were <10 years old but also those in our age range criteria (aged 10–19 years) were also included.

However, studies that reported resilience interventions in the context of pathology [e.g., somatic (cancer, chronic illness) or serious mental disease (such as schizophrenia)] were excluded.

### Search methods

The search strategy was carried out in July 2021 in four separate databases: PubMed, EMBASE, PsycINFO, and Web of Science. Keywords were translated to MeSH terms, using an “entry term” to check the synonyms and “equivalence relations” for the extension of the search. The search strategy included MeSH terms and keywords in case there was not a specific MeSH term for the search term needed. The search was limited to the last 10 years to review the most recent interventions.

The search strategy included the following MeSH terms and keywords (MEDLINE/PUBMED): “(((((((((“Parent-Child Relations”[Mesh]) OR “Interpersonal Relations”[Mesh]) OR “Social Participation”[Mesh]) OR “Students”[Mesh]) OR “Emotional Regulation”[Mesh]) OR “Empathy”[Mesh]) OR “Self Concept”[Mesh]) OR “Adaptation, Psychological”[Mesh] OR (“Social Support”[Mesh])) AND ((“Resilience, Psychological”[Mesh] OR resilience) AND (“Psychosocial Intervention”[Mesh] OR intervention)). A detailed overview of the search strategy for all four databases is presented in Supplementary material 2.

### Study selection

The study selection and data extraction were carried out by two independent reviewers (MLL and BGF). The search strategy was carried out, and all references were imported to the Rayyan screening tool (Ouzzani et al., 2016). Duplicates were excluded. The two independent reviewers selected the studies independently according to the eligibility criteria. The reasons for excluding studies during the full-text phase were recorded. Any disagreement regarding the eligibility of the studies was resolved through discussion or by referring to a third researcher (LM). The final list of included studies was also verified by two reviewers.

### Data collections

We designed an *ad hoc* data extraction matrix to record the following data: (a) characteristics of the publication (year, author, and country of study); (b) intervention programme (name); (c) sample size and sociodemographic characteristics (age, risk context); (d) study design; (e) intervention details (focus, duration, number of sessions, and content); (f) resilience measures (g) qualitative and quantitative results of primary and secondary measures; (h) quality assessment indicators; and (i) duration of follow-up. Data were extracted by three researchers (MLL, BGF, and LM).

### Quality assessment

The risk of bias was assessed by two independent researchers (MLL and BGF). The risk of bias in the included studies was assessed using the Cochrane risk of bias tool (Higgins et al., 2011). This tool checks potential sources of bias, including the adequate generation of the allocation sequence; the concealment of allocation to treatment conditions; blinding of personnel and participants; blinding of outcome assessors; handling of incomplete data; selective outcome reporting; and other possible risks of bias. The risk of bias was classified as high, low, and unclear.

Participant and personnel blinding were assessed, and it was considered a high risk given the difficulty in masking any condition groups for participants and personnel. All studies were included regardless of their quality.

### Meta-analysis

The meta-analysis was estimated for the continuous variables, calculating the standardised mean difference (SMD) with 95% confidence intervals (CIs). SMD was chosen because the identified studies used different quantitative scales to measure resilience. A random-effect meta-analysis was used to obtain pooled estimates because of the population heterogeneity, setting, and duration of the intervention. The estimation of SMD was performed by applying Cohen's *d* approach (Cohen, 1988). The confidence intervals for the random effects estimate were calculated based on standard normal quantile (DerSimonian and Laird, 1986), and the DerSimonian-Liard estimator was used to estimate the between-study variance. The prediction interval (PI), used to estimate the true effect size and plot a distribution of true effects, was also calculated using the bootstrap approach proposed by Nagashima et al. (2019). Given that we expected differences in effect size, stratified analyses were done according to (a) population type (general, at risk); (b) study design; (c) intervention type [multicomponent interventions, social and emotional learning or similar counselling/mentoring, mindfulness, cognitive behavioural therapy (CBT)]; and (d) age range (early and middle adolescence). Heterogeneity was measured by the Higgins test (*I*^2^). A value of 0–40% indicated low heterogeneity, 40–75% moderate, and 75–100% considerable heterogeneity (Higgins and Thompson, 2002). The results were considered statistically significant if the *p*-value was < 0.10. Publication bias was assessed by visual inspection of the funnel plots and through the Egger test.

There were several elements of heterogeneity among the studies assessing the effectiveness of resilience interventions (Chmitorz et al., 2018). Principal among them were the lack of a resilience definition, the different scales used for assessing resilience, and the use of surrogate outcomes for measuring the interventions. In addition, there were also differences in follow-up assessments. To account for potential variations that could alter the outcome of the meta-analysis, we conducted sensitivity analyses on factors such as study quality, based on the Cochrane tool, and intervention duration, using the Cochrane tool to assess each item individually. While the statistical analyses were performed using R Statistical Software (v. 4.3.0; R Core Team, 2023) and Stata v.15.1, the meta-analysis was conducted using the Meta R package (v6.5.0; Balduzzi et al., 2019).

## Results

### Search outcome

**Figure 1:**
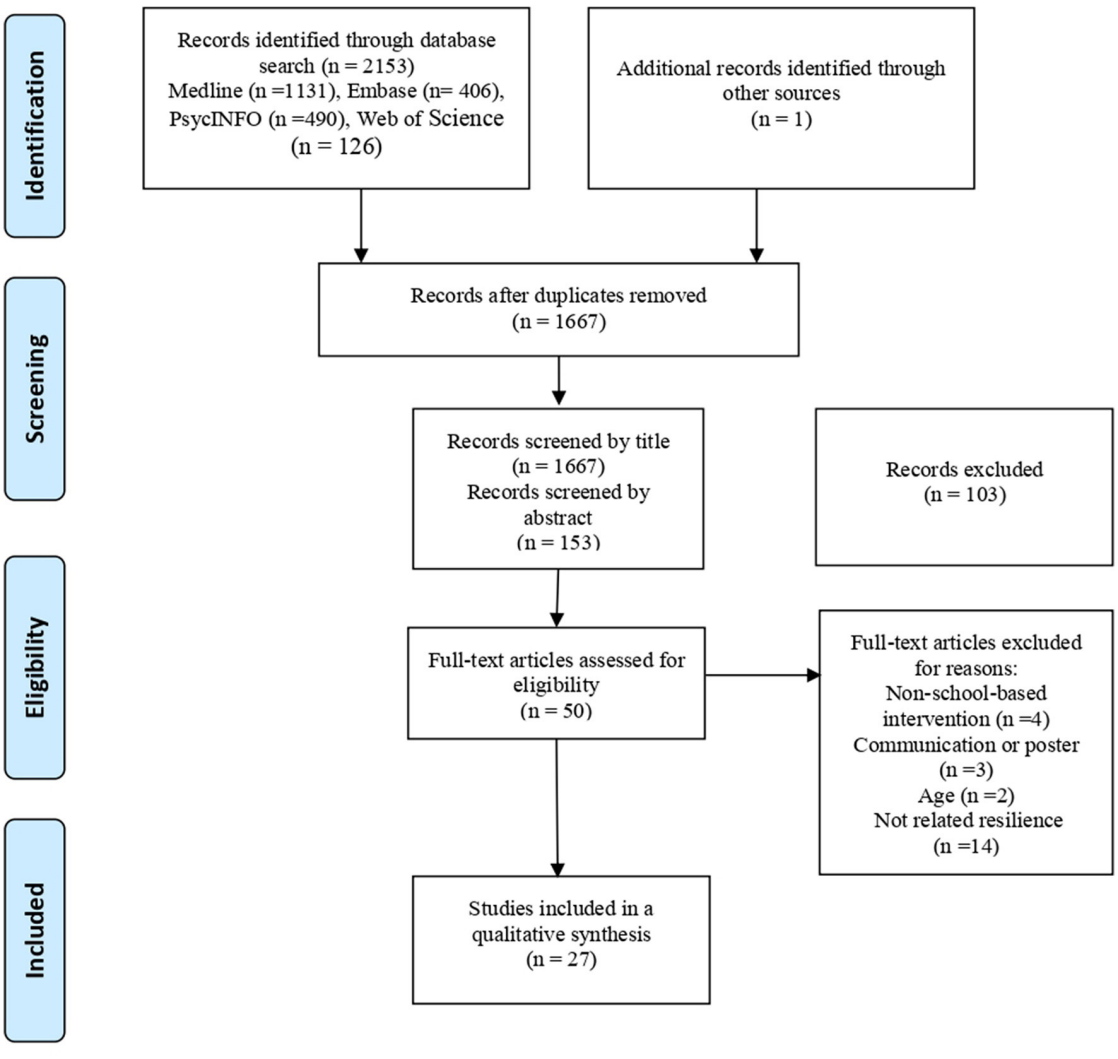
PRISMA flow diagram—systematic review of effectiveness of resilience-based interventions in schools for adolescents.

The search strategy resulted in a total of 1,667 publications after excluding duplicates, which were screened according to the eligibility criteria by title, resulting in the selection of 153 articles. They were further filtered by abstract, resulting in the selection of 50 articles. After a full-text review, 27 articles were eventually selected for the final review. The reasons for excluding articles after full-text review were as follows: 14 trials had outcomes that were not related to resilience; four were not school-based interventions; three were communications or posters; and two included non-adolescents. The PRISMA flow diagram of the study's selection process is shown in Figure 1.

Of the 27 articles included in this study (Hyun et al., 2010; Fu et al., 2013; Slone et al., 2013; Castro-Olivo, 2014; Chen et al., 2014; Leventhal et al., 2015; Chisholm et al., 2016; Gudiño et al., 2016; Ruttledge et al., 2016; Furness et al., 2017; Ijadi-Maghsoodi et al., 2017; Sarkar et al., 2017; Scarf et al., 2017; Stapleton et al., 2017; McAllister et al., 2018; Mirza and Arif, 2018; Osofsky et al., 2018; Felver et al., 2019, 2020; Kuperminc et al., 2020; Maalouf et al., 2020; Sugiyama et al., 2020; Suranata et al., 2020; Tripa et al., 2020; Volanen et al., 2020; Kelley et al., 2021; Zhang et al., 2021), 9 (33.3%) studies were RCTs, 2 (7.4%) were clustered RCTs (cRCTs), 12 (44.4%) were quasi-experimental, 2 (7.4%) were pre-post that the outcome was measure before and after the intervention studies, and 2 (7.4%) used mixed-methods. The articles included a total of 8,591 adolescents from the general population and 7,324 adolescents exposed to risk (Table 1). Only 18 studies assessed risk contexts as follows: affected by natural disasters (Hiroshima heavy rain, Katrina hurricane, and earthquake) (*n* = 4); belonging to disadvantaged minority ethnic groups (*n* = 4); living in rural and remote areas (*n* = 2); parental alcohol abuse disorders (*n* = 1); anxiety problems (*n* = 1); left-behind children (*n* = 1); low or moderate resilience (*n* = 1); low self-efficacy (*n* = 1); non-resilient at risk of failure (*n* = 1); armed conflict (*n* = 1); and non-specific high-risk group (*n* = 1).

**Table 1:** Characteristics of the studies included in the systematic review of the effectiveness of resilience-based interventions for adolescents in schools.

**References**	**Intervention name**	**Location**	**Participant's *n*/age/context risk (general population, war)**	**Design**	**Intervention sessions (s)/weeks (w) duration (min)**	**Intervention focus/protective factors**	**Resilience measure**
Kelley et al. (2021)	Innate Health Education and Resilience Training (iHEART)	London, United Kingdom	269 students (aged 11–15). General population. EG (*n* = 205); CG (*n* = 64).	Mixed-methods; quasi-experimental	SEL 2 facilitators 10S, 1S weekly 50 min	Human psychological system; and real-life issues (stress, anxiety, bullying, and social media)	The Inside-Out Resilience Questionnaire (I-ORQ)
Tripa et al. (2020)	SEL and mindfulness programme	Romania	62 left-behind children (aged 12–15). Mean age 12.71 (*SD* = 1.868). EG (*n* = 31); CG (*n* = 31).	Quasi-experimental	SEL and mindfulness 1 experiment 6S, 1S weekly 90 min	Self-awareness; awareness of others; problem-solving; decision making; and flexibility	Brief Resilience Scale (BRS); Strengths and Difficulties Questionnaire (SDQ)
Zhang et al. (2021)	Psychological Counselling	Shandong, China	160 adolescents (aged 12–18) with anxiety symptoms. EG (*n* = 80); CG (*n* = 80).	Quasi-experimental	Counselling; RT 8S, 1S weekly 60 min Sport Programme Expert 16 S/8 w 50 min	Physical activity; positive emotions; tolerance; perceive gratitude; coping skills; and problem-solving	Healthy Kids Resilience Assessment
Maalouf et al. (2020)	FRIENDS	Lebanon	280 (aged 11–13). General population. EG (*n* = 145); CG (*n* = 135).	RCT	SEL RT (MH) 10S, 1S weekly 45–50 min	Understanding feelings; empathy; learning to relax; challenging one's thoughts; problem-solving; and making and retaining friends	Strengths and Difficulties Questionnaire (SDQ)
Felver et al. (2020)	Kripalu Yoga in Schools (KYIS)	New York, United States	23 students. Mean age 12.1 years. General population. EG (*n* = 9); CG (*n* = 14).	Quasi-experimental	1 Yoga instructor 15S, 8w 45 min	Social-emotional competencies: self-awareness, physiological response to stress, and compassion for the self and others	Social-Emotional Assets and Resilience Scales (SEARS)
Sugiyama et al. (2020)	CBT	Japan	229 second-grade senior students affected by the Hiroshima heavy rain disaster. EG (*n* = 229).	Pre-post	CBT 1 Psychologist 1S 50 min	Psychological education and problem-solving on daily stress and traumatic experiences	Tachikawa Resilience Scale (TRS)
Suranata et al. (2020)	Konseling Remaja	Bali, Indonesia	90 students (aged 12) (Low or moderate resilience). EG “Face to face” (*n*= 30); EG “Internet-based” (*n* = 30); CG (*n* = 30).	RCT	Face-to-face counselling 3 counsellors 8S, 1S weekly 55 min Internet-based counselling 3 w. 30 min	Social skills; relaxation; problem-solving; and assertiveness	Psychological subscale of Resilience Youth Development Module (RYDM)
Kuperminc et al. (2020)	Project Arrive (PA)	Western United States	Students at high risk. Mean age 14 years (12.8–15.9). EG (*n* = 114); CG (*n* = 71).	Quasi-experimental	Mentoring Sessions with co-mentors for 50 min over a full academic year	Self-efficacy; empathy; problem-solving; self-awareness; school belonging; school support; school meaningful participation; peers; home support; and home meaningful participation	Resilience Youth Development Measure (RYDM)
Volanen et al. (2020)	The Healthy Learning Mind (HLM). “Skills for Wellbeing”	Finland	3,519 students (aged 12–15). General population. EG (*n* = 1,646); CG active (*n* = 1,488); CG inactive (*n* = 385).	Cluster RCT	Mindfulness 1 facilitator 9S, 1S weekly 45 min	Emotional awareness; sustained attention and attention; and emotional regulation	The Resilience Scale (RS14); Strengths and Difficulties Questionnaire (SDQ)
Felver et al. (2019)	Learning to BREATHE	New York, United States	27 students at risk (ethnically diverse). Mean age 16.39 (*SD* = 1.04). EG (*n* = 16); CG (*n* = 11).	RCT	Mindfulness RT 7 S/9 w 48 min	Awareness and emotions	Social-Emotional Assets and Resilience Scales (SEARS)
McAllister et al. (2018)	iCARE-Rural	Queensland, Australia	850 young people from rural and regional areas (ages 11–14). Mean age 13 years (*SD* = 0.55). EG (*n* = 850).	Pre-post	Mental Health Promotion Programme. Nurses, guidance officers, and teachers 6 S/6 w	Good care; problem-solving; meditation; self-efficacy; and good for others (altruism)	The General Self-Efficacy (GSE); The Social Emotional Assets and Resilience Scale (SEARS)
Mirza and Arif (2018)	Fostering Academic Resilience	Pakistan	64 students, non-resilient and at risk of failure (aged 14–16). EG (*n* = 32); CG (*n* = 32).	RCT	Foster protective factors Resilience teacher 12 S/12 w 60 min	Creativity; internal locus of control; self-concept; self-esteem; self-efficacy; autonomy; sense of purpose in life; optimism; sense of humour; and teacher-student relationship	Resilience Assessment Scale (RAS)
Osofsky et al. (2018)	The Youth Leadership Programme (YLP)	Louisiana, United States	214 students affected by natural disasters (Hurricane Katrina) (aged 15–17). EG (*n* = 71); CG (*n* = 143).	Quasi-experimental	Leadership P. Teacher and mental health professional. Meeting weekly during lunch period; Summer YLP (1 month); YLP Leadership Summit	Self-efficacy; engaging community members; self-awareness; and leadership	The Status Questionnaire for Students
Furness et al. (2017)	Positive Youth Development Programme Project K	New Zealand	80 students with low self-efficacy (aged 13–15). EG (*n* = 49); CG (*n* = 31).	Quasi-experimental	Adventure education/mentoring 3 w residential wilderness; 10-day community challenges; 12-month mentoring partnerships	Wilderness adventure: goal setting, teamwork, problem-solving, and leadership; community challenge: community services, citizenship, and academic outcomes; individual mentoring: emotional support, role model, and academic outcomes	25-item Resilience Scale (RS); Project K Self-efficacy (PKSEQ)
Ijadi-Maghsoodi et al. (2017)	The Resilience Classroom Curriculum	United States	100 students among low-income racial and ethnic minorities (aged 14–15) EG (*n* = 100).	Mixed-methods (pre-post and focal group)	SEL Social workers/teachers 9S/ 9 w or month 45–55 min	Social-emotional skills; school climate; problem-solving; goal-setting; emotional regulation; communication; and managing stress	Resilience Youth Development Module (RYDM)
Sarkar et al. (2017)	Life Skills Empowerment	West Bengal, India	742 students. Mean age 13.5 years (*SD* = 1.53). Tribal/non- tribal areas EG (*n* = 381); CG (*n* = 361).	Quasi-experimental	Life skills education 2 trainers 2S/ 1 w 45–120 min	Basic life skills; motivation; discipline; nutrition; health and hygiene; relationship; self-awareness; sexuality; and social responsibility	Child Youth Resilience Measure (CYRM-28)
Scarf et al. (2017)	Adventure Education Programme (AEP)	New Zealand	270 adolescents (aged 15–19). General population. EG (*n* = 180); CG (*n* = 90).	Quasi-experimental	Adventure education 10 days voyage on Spirit of New Zealand	Social connectedness; social support; and a sense of belonging	Resilience Scale (RS)
Stapleton et al. (2017)	Emotional Freedom Techniques (EFT)	Australia	204 students (aged 14–16). General population. EG (*n* = 80); CG (*n* = 124).	Quasi-experimental	EFT, clinical psychologist and psychotherapist 5 S/5 w 75 min	Fears and anxiety; negative self-statements; academic and sporting performance; expectations of themselves and others; and goal settings	Connors-Davidson Resilience Scale (CD-RISC); Strengths and Difficulties Questionnaire (SDQ)
Chisholm et al. (2016)	SchoolsSpace	Birmingham, United Kingdom	769 students (aged 11–13). General population. EG (*n* = 405); CG (*n* = 364).	Cluster RCT	Contact intervention 1-day mental health professional and young person with mental illness	Stigma of mental illness; Stress and anxiety; different ways of thinking; and thoughts, feelings, and behaviours	15-item Resilience Scale (RS); Strengths and Difficulties Questionnaire (SDQ)
Gudiño et al. (2016)	Skills Training in Affective and Interpersonal Regulation (STAIR-A)	New York, United States	46 racial/ethnic minority girls (aged 11–16). EG (*n* = 23); CG (*n* = 23).	Quasi-experimental	STAIR 2 doctoral-level therapists 16 S/1 semester	Emotional regulation and interpersonal competencies; social support; and a sense of control	Resilience factors: subscales from BASC-SRP
Ruttledge et al. (2016)	FRIENDS for Life	Ireland	709 children (aged 9–13). EG (*n* = 333); CG (*n* = 376). General population.	RCT	CBT School teachers 10 S/10 w.	Feelings; relaxation; self-talk; coping; problem-solving; skills to help oneself and others	Coping Efficacy Scale
Leventhal et al. (2015)	Girls first resilience curriculum	Bihar, India	2,548 adolescent girls. Middle-income countries Mean age 12.99 years (*SD* = 1.17). EG (*n* = 1,752); CG (*n* = 756).	RCT	Resilience curriculum Masters trainers 23 S/23 w 60 min	Listening skills; character strengths; life stories and goals; identifying emotions; problem-solving; forgiveness and apologies; and pace projects	Connor-Davidson Resilience Scale−10. (CD-RISC); Child and Youth Resilience Measure-28 (CYRM-28); General Self-Efficacy Scale (GSE)
Castro-Olivo (2014)	*Jóvenes Fuertes* (Strong Teens)	United States	102 adolescents, Latino. Mean age 13.91 years (*SD* = 1.86). EG ( *n*= 49); CG (*n* = 53).	Quasi-experimental	SEL 2 Latino master's level teachers 12 S/12 w	Self-awareness; social awareness; empathy; problem-solving; anger management; responsible decision making; and goal setting	BERS-2
Chen et al. (2014)	Short-term CBT	Sichuan, China	40 adolescents who lost at least one parent in an earthquake. Mean age 14.50 (*SD* = 0.71). EG (*n* = 16); CG: support group (*n* = 12); non-intervention group (*n* = 12).	RCT	CBT Trained MH professional 6 S/6 w 60 min	Narrative and learning skills to cope with PTSD. Relaxation and coping	Connor-Davidson Resilience Scale (CD-RISC)
Fu et al. (2013)	A Sports-Based Psychosocial Intervention to Foster Resilience	China	4,120 child and adolescent survivors of the 2008 earthquake (aged 6–16). EG (*n* = 1,988); CG (*n* = 2,132).	Quasi-experimental	Sport programme Caregivers Once or twice a week (September 2008-July 2009) 45 min	Coping skills; moving forward: self-esteem, communication skills	The Connor-Davidson Resilience Scale (CD-RISC)
Slone et al. (2013)	School-Based Primary Prevention Intervention	South Israel	179 adolescents were war exposed. Mean age 16.3 years (*SD =* 1.1). EG (*n* = 94); CG (*n* = 85).	RCT	Handbook (mobilisation of support and self-efficacy) Teachers Six activities during lessons in classrooms. Twice weekly for 3 weeks	Social support; cooperation; social and community involvement; times to be alone and time to be with others; self-efficacy; coping; problem-solving; self-relaxation; self-control; personal strength; and self-empowerment	Self-Efficacy Questionnaire for Children (SEQ-C); Strengths and Difficulties Questionnaire (SDQ) Social Support Matrix
Hyun et al. (2010)	CBT	South Korea	34 male at-risk adolescents (alcohol-dependent parents). EG (*n* = 17); CG (*n* = 17).	RCT	CBT 2 RT 10S, 1S weekly 50 min	Self-concept; self-esteem; stress management; dysfunctional coping; and healthy coping strategies	Korean Adolescents Resilience Scale

EG, experimental group; CG, control group; SEL, social and emotional learning; S, sessions; w, weeks; RT, research team; MH, mental health; P, programme; CBT, cognitive-behavioural therapy. RCT, randomized control trial.

A total of 7 (25.9%) studies were undertaken in the United States, 3 (11.1%) in India and China, and 2 (7.4%) in Australia, the United Kingdom, and New Zealand. Other countries in which a single study was carried out were Finland, Indonesia, Ireland, Israel, Japan, Lebanon, Pakistan, and South Korea.

### Type of interventions

Of the 27 studies that were included, 11 (40.7%) involved multicomponent interventions based on more than one technique [mindfulness, social, and emotional learning (SEL); counselling; skills training in affective and interpersonal regulation; mental health promotion programme; foster protective factors; leadership programme; life skills education; resilience curriculum; sports programme; adventure programme; handbook for mobilisation of support; and self-efficacy]. Further, 5 (18.5%) studies were based on social and emotional learning or similar (emotional freedom techniques) interventions; 4 (14.8%) were interventions based on CBT; 3 (11.1%) on mindfulness or yoga; 3 (11.1%) on counselling or mentoring; and 1 (3.7%) on contact intervention. The intervention duration ranged from a full academic year to 1-day sessions (Table 1). The number of sessions and the duration of the interventions were specified in 20 and 17 studies, respectively. The number of sessions ranged from 1 to 23, with a mean of 9.25. The mean duration of the sessions was 59.33 min.

### Resilience focus on intervention

Fifty-six interventions focused on protective factors of resilience (Table 1). Those related to individual skills were as follows:

(a) Behaviour: managing stress/anxiety (9); coping skills (6); leadership (2); meditation (2); attention (1); autonomy (1); discipline (1); flexibility (1); caring (1); moving forward (1); and physical activity (1).
(b) Cognitive: problem-solving (13); self-awareness (7); self-efficacy (5); goal setting (5); learning to relax (5); a sense of control, internal locus of control (4); character strengths (2); decision making (2); human psychological system (2); academic outcomes (1); challenging one's thoughts (1); competence (1); creativity (1); self-empowerment (1); sense of purpose in life (1); and stigma of mental health (1).
(c) Communications skills (3).
(d) Emotional: social-emotional competence (positive emotions, emotional awareness, emotional regulation), identifying emotions and feelings, anger management, negative self-statements (12); empathy, solidarity, altruism, compassion, tolerant for others (10); social skills (4); self-esteem (3); awareness of others (2); self-concept (2); expectative of themselves and others (1); motivation (1); optimism (1); perceived gratitude (1); a sense of belonging (1); a sense of humour (1); and social responsibility (1).

The focus of the interventions related to the environmental dimension were as follows:

(a) Family: home support (1) and home meaningful participation (1).
(b) Peers: relationship (3) and teamwork (1).
(c) Community: engaging community members (3); social support (3); emotional support (1); role model (1); social connectedness (1); and social media (1);
(d) School: school support (2); school belonging (1); school climate (1); School participation (1); and teacher relationship (1).

### Resilience measures and assessments

Resilience was evaluated using 21 different measures. Ten (37.0%) studies used more than one scale to assess resilience. The main scales found in the literature were the following: the Strengths and Difficulties Questionnaire (SDQ) in six studies (22.2%); the Connor-Davidson Resilience Scale (CD-RISC), and the Resilience Scale in four studies (14.8%); Resilience Youth Development Measure (RYDM) and the Social-Emotional Assets and Resilience Scale (SEARS) in three studies (11.1%); and the Child Youth Resilience Measure (CYRM−28) and The General Self-Efficacy (GSE) in two studies (8.6%) (Table 1).

In 26 studies, an assessment was conducted immediately after the intervention and in 1 study, the assessment was undertaken 2 weeks after follow-up. A baseline assessment was conducted in 26 studies, and in nine studies, a follow-up assessment was also undertaken. The follow-up period ranged from 2 weeks to 12 months. Only 4 (14.8%) studies exclusively evaluated resilience. Other outcomes were assessed in 23 studies (85.1%); most of them (55.5%) were related to psychological symptoms or mental health problems (depression, anxiety, or post-traumatic stress disorder), and 11.1% were behavioural problems. A significant increase in resilience was reported in 20 (74%) studies, and 8 (29.6%) studies reported a significant reduction in mental health problems and psychological symptoms (anxiety, depression, and trauma). Only two studies reported no significant outcomes (Table 2).

**Table 2:** Overview of the study measures, assessments, and outcomes.

**References**	**Measures**	**Pre-asses**	**Post-asses**	**Follow-up**	**Outcome**
Kelley et al. (2021)	Mental wellbeing, psychological resilience	✓	✓	–	Resilience and wellbeing
Tripa et al. (2020)	Resilience, loneliness, and social dissatisfaction		✓	–	Self-regulation of abilities, difficulties, and social dissatisfaction
Zhang et al. (2021)	Resilience, depression, and anxiety	✓	✓	–	Addressed emotional disorders (anxiety and depression), sleep quality, and psychological resilience
		✓	✓		
Maalouf et al. (2020)	Resilience, depression, and anxiety symptoms	✓	✓	–	General emotion and depressive symptoms addressed
Felver et al. (2020)	Behavioural problems and social-emotional competence	✓	✓	–	Social-emotional competence
Sugiyama et al. (2020)	Depression and resilience	✓	✓	–	Depression and resilience
Suranata et al. (2020)	Resilience	✓	✓	5 weeks	Resilience
Kuperminc et al. (2020)	External and internal resilience	✓	✓	–	External resilience resources
Volanen et al. (2020)	Resilience, socioemotional functioning, and depressive symptoms	✓	✓	26 weeks	Resilience, depressive symptoms, and socioemotional functioning
Felver et al. (2019)	Psychosocial resiliency and psychosocial problem behaviour	✓	✓	–	Resilience
McAllister et al. (2018)	Resilience, coping, and self-efficacy	✓	✓	8 weeks	Self-efficacy and coping strategies
Mirza and Arif (2018)	Resilience	✓	✓	–	Academic resilience
Osofsky et al. (2018)	Resilience (self-efficacy) and trauma symptoms	✓	✓	–	Self-efficacy and trauma
Furness et al. (2017)	Self-efficacy, resilience, connectedness, and wellbeing	✓	✓	–	Self-efficacy and resilience
Ijadi-Maghsoodi et al. (2017)	Resilience, post-traumatic stress disorder (PTSD), and school support	✓	✓	–	Resilience
Sarkar et al. (2017)	Resilience, internal health locus of control, self-determination, and pathological behaviour	✓	✓	12 weeks	Resilience, internal health locus of control and self-determination, and pathological behaviours
Scarf et al. (2017)	Resilience, social support, and a sense of belonging	✓	✓	–	Resilience
Stapleton et al. (2017)	Resilience, self-esteem, fear of failure, and strengths and difficulties	✓	✓	12–48 weeks	Fear of failure and emotional and behavioural difficulties
Chisholm et al. (2016)	The stigma of mental illness, knowledge of mental illness, emotional wellbeing, resilience, and help-seeking	✓	–	2 weeks	Stigma of mental illness and wellbeing
Gudiño et al. (2016)	Resilience: social stress, interpersonal relationships, locus of control, symptoms of psychopathology (PTSD, depression, and anxiety symptoms	✓	✓	3 months	Social engagement and locus of control and depressive symptoms
Ruttledge et al. (2016)	Resilience (coping), self-concept, school connectedness, anxiety symptoms, wellbeing, and social validity	✓	✓	2 months	Wellbeing, general coping skills, and a sense of connectedness with school
Leventhal et al. (2015)	Emotional resilience, self-efficacy, social-emotional assets, psychological and social wellbeing, depression, and anxiety symptoms	✓	✓	–	Emotional resilience, self-efficacy, social-emotional assets, psychological, and social wellbeing
Castro-Olivo (2014)	Social-emotional outcomes and resilience	✓	✓	–	Resilience
Chen et al. (2014)	Psychological resilience, symptoms of PTSD, and depression	✓	✓	3 months	PTSD, depression symptoms, and psychological resilience
Fu et al. (2013)	Resilience and PTSD	✓	✓	–	PTSD
Slone et al. (2013)	Resilience (mobilisation of support and self-efficacy), and psychological symptoms	✓	✓	–	Self-efficacy and psychological symptoms
Hyun et al. (2010)	Resilience, self-concept, and depression symptoms	✓	✓	–	Resilience

A systematic review of the effectiveness of resilience-based interventions for adolescents in schools.

### Risk of bias in the included studies

**Figure 2:**
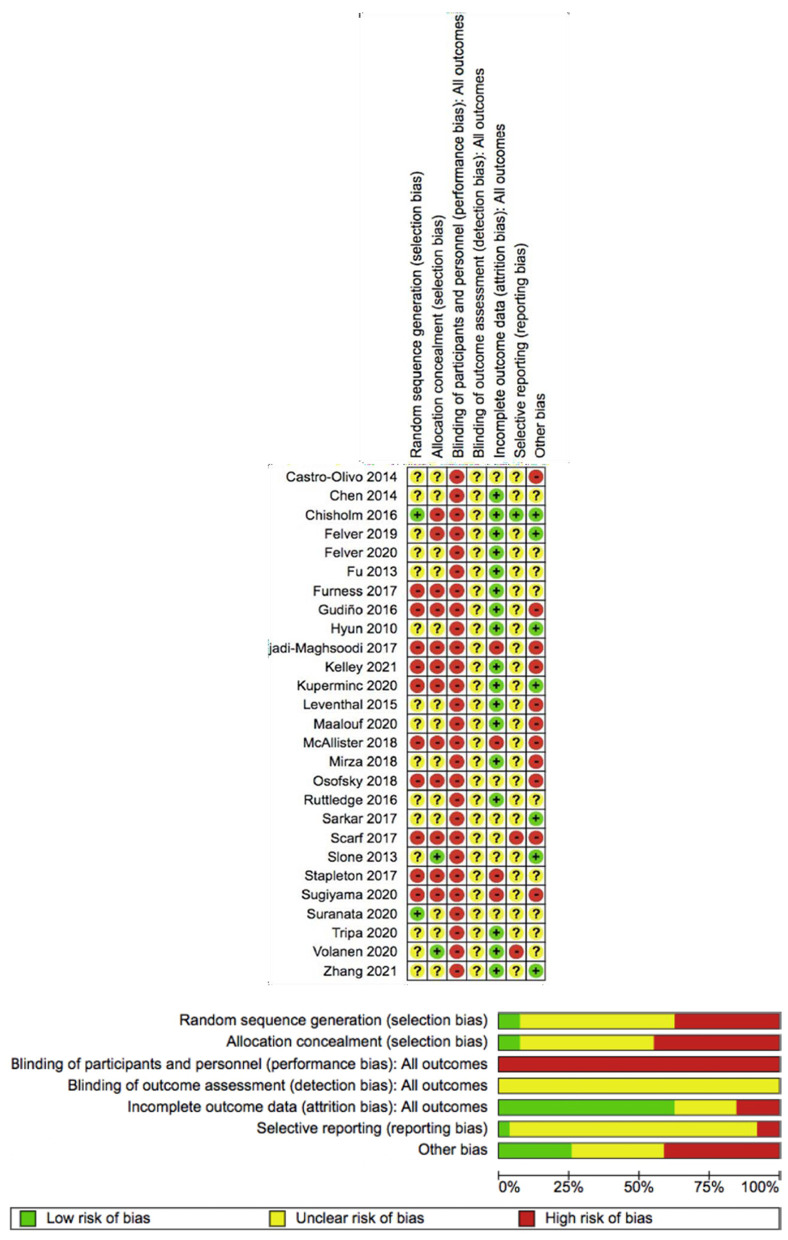
Risk of bias assessment of the included studies—systematic review of the effectiveness of resilience-based interventions in schools for adolescents.

The risk of bias was determined across all domains of the Cochrane collaboration tool. Two (7.4%) studies reported adequate sequence generation and 15 (55.6%) were unclear. Two studies reported adequate allocation concealment and 12 (21.8%) were unclear. All the studies reported high-risk blinding of participants and personnel, making it difficult to mask any condition groups. In the 27 articles we reviewed, the blinded outcome assessment was unclear; there was not enough information to verify the outcomes. Seventeen studies reported complete outcome data. Only two trials had an intervention protocol to assess the risk of reported bias, and in one of them, only primary outcomes were reported. Eleven studies showed a high risk of bias for other causes. For instance, there was no control group in three studies; the sample only included male or female subjects in three studies; there were differences in the baseline characteristics of the sample in two studies; and the scale to assess the outcomes was not validated, participants were not well-reported, and not enough information was reported on the characteristics of the experimental group or the control group in one study (Figure 2).

### Efficacy of resilience in school-based interventions

#### Meta-analysis

**Figure 3:**
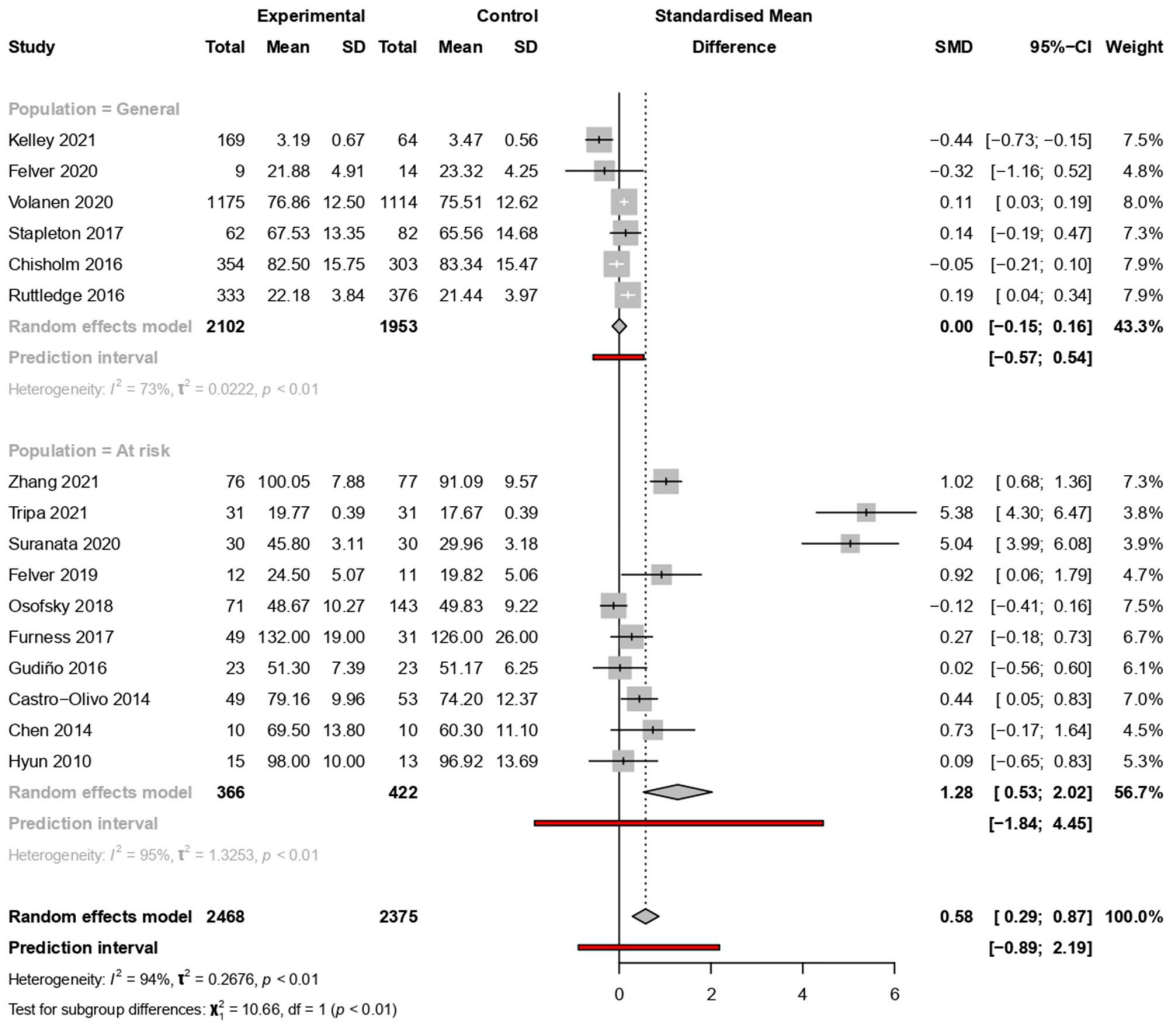
Meta-analysis of the effectiveness of school-based interventions for adolescents.

From the 27 studies included in this review, 11 were excluded from the meta-analysis—seven did not provide the necessary statistical data for meta-analysis (Fu et al., 2013; Slone et al., 2013; Leventhal et al., 2015; Sarkar et al., 2017; Scarf et al., 2017; Kuperminc et al., 2020; Maalouf et al., 2020), three had no control group (Ijadi-Maghsoodi et al., 2017; McAllister et al., 2018; Sugiyama et al., 2020), and one had consistent outliers in the forest plot, probably due to its large mean value (Mirza and Arif, 2018). Thus, 16 studies were finally included in the meta-analysis. Of those that included results from more than one measurement scale, we selected the instruments that were specifically developed for measuring resilience. These included the following: interpersonal relations (Gudiño et al., 2016), a 14-item resilience scale (Volanen et al., 2020), a 25-item resilience scale (RS-25) (Furness et al., 2017), the Connors-Davidson Resilience Scale (CD-RISC) (Stapleton et al., 2017), and a 15-item resilience scale (Chisholm et al., 2016). The random effects of SMD indicated an overall increase in resilience after the intervention [SMD = 0.58, 95% CI (0.29–0.87)]. Predictive interval (PI) ranged from −0.85 to 2.18. There was high heterogeneity among studies (*I*^2^ = 94%, *p* < 0.01) (Figure 3).

### Subgroup analysis

#### Population type

The intervention effects were analysed according to the population subgroups. Six studies provided information about the general population (*n* = 5,493). Ten studies included the population at risk (*n* = 855), which comprised minority ethnicity (three studies); those affected by natural disasters (two studies); those whose parents had alcohol abuse disorders (one study); those with anxiety symptoms (one study); left-behind children (1 study); those with low or moderate resilience (one study); and those with low self-efficacy (one study). There was a significant increase in resilience among the population at risk [SMD = 1.28, 95% CI (0.53–2.02), PI (−1.84 to 4.45)] with considerable heterogeneity (*I*^2^ = 95%, *p* < 0.01), but not in the general population [SMD = 0.00, 95% CI (−0.15 to 0.16), PI (−0.57 to 0.53)] (Figure 3).

#### Study design

**Figure 4:**
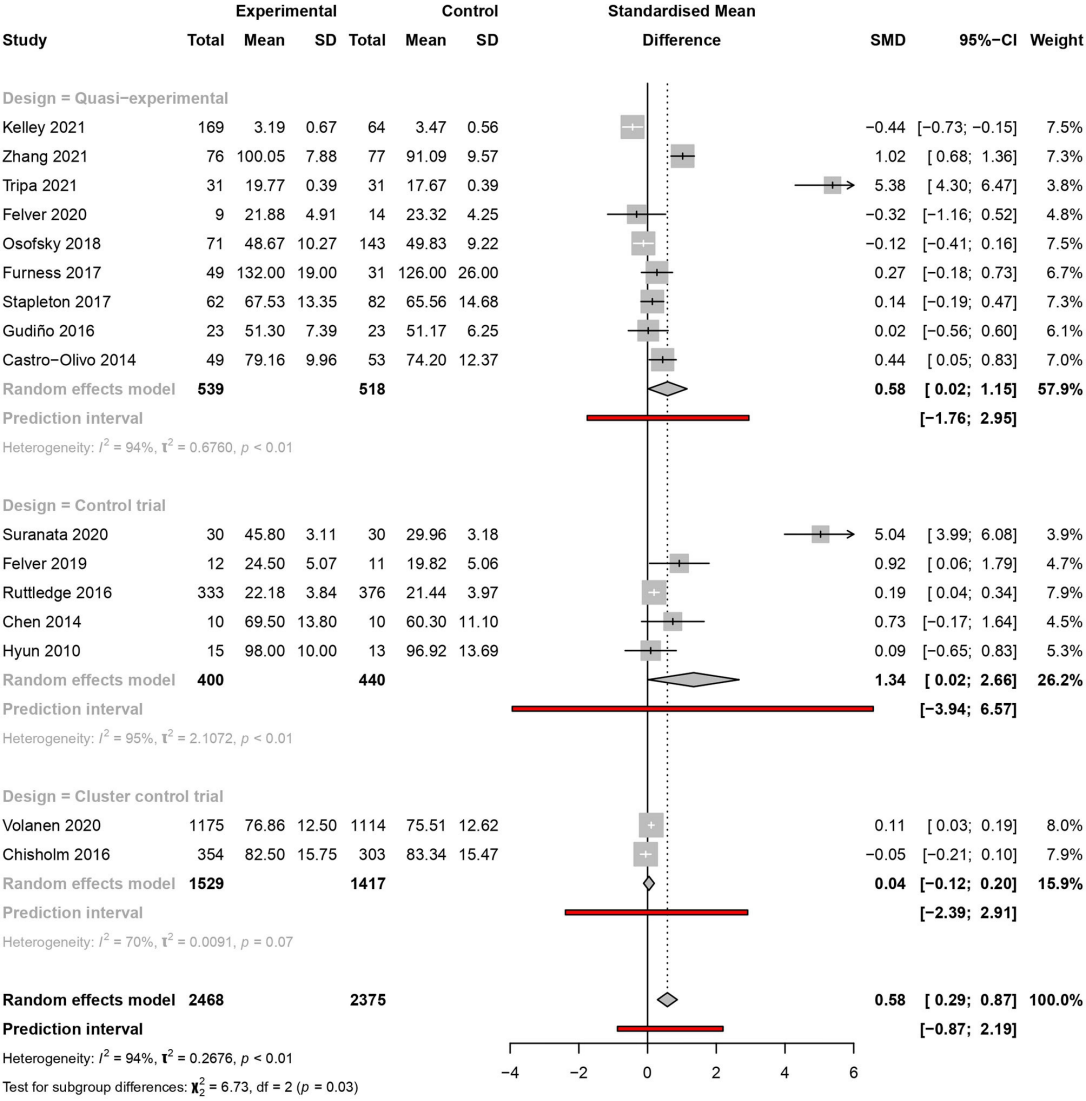
Meta-analysis of the effectiveness of school-based interventions for adolescents according to study design.

The meta-analysis of nine quasi-experimental studies showed an increase in resilience with considerable heterogeneity [SMD = 0.58, 95% CI (0.02–1.15), PI (−1.76 to 2.95)] (*I*^2^ = 94%, *p* < 0.01). Similar results were observed for RCTs [SMD = 1.34, 95% CI (0.02–2.66), PI (−3.94 to 6.57)] (*I*^2^ = 95%, *p* < 0.01) (Figure 4).

#### Intervention type

**Figure 5:**
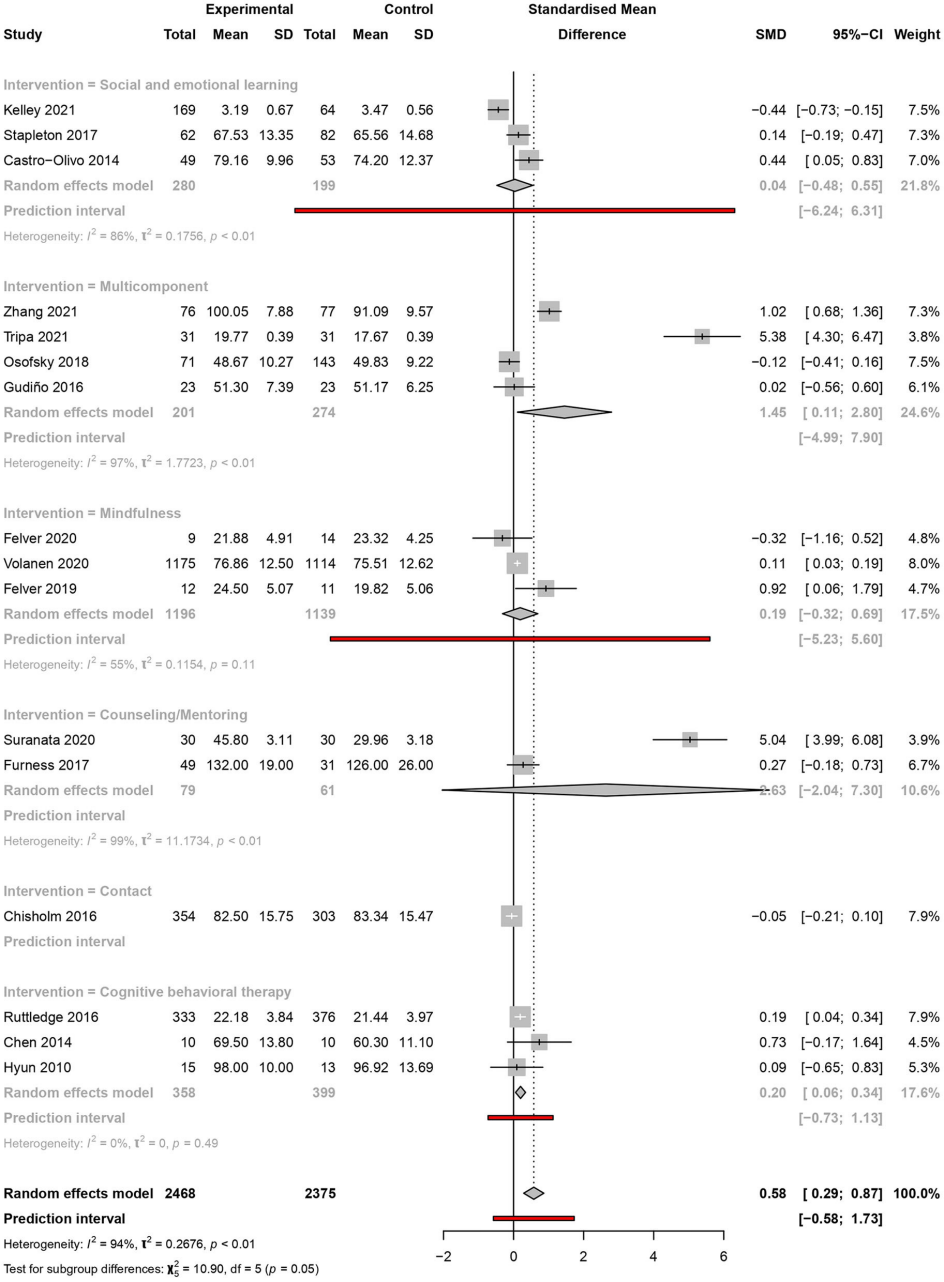
Meta-analysis of the effectiveness of school-based interventions for adolescents according to intervention type.

From the selected studies, four analysed multicomponent interventions; three assessed social and emotional learning interventions, mindfulness, and CBT; two assessed counselling or mentoring-based interventions; and one study focused on contact interventions. Resilience was significantly increased only in the multicomponent [SMD = 1.45, 95% CI (0.11–2.80), PI (−4.99 to 7.90)] (*I*^2^ = 97%, *p* < 0.01) and CBT interventions [SMD = 0.20, 95% CI (0.06–0.34), PI (−0.73 to 1.14)] (*I*^2^ = 0%, *p* < 0.01) (Figure 5).

#### Age range

**Figure 6:**
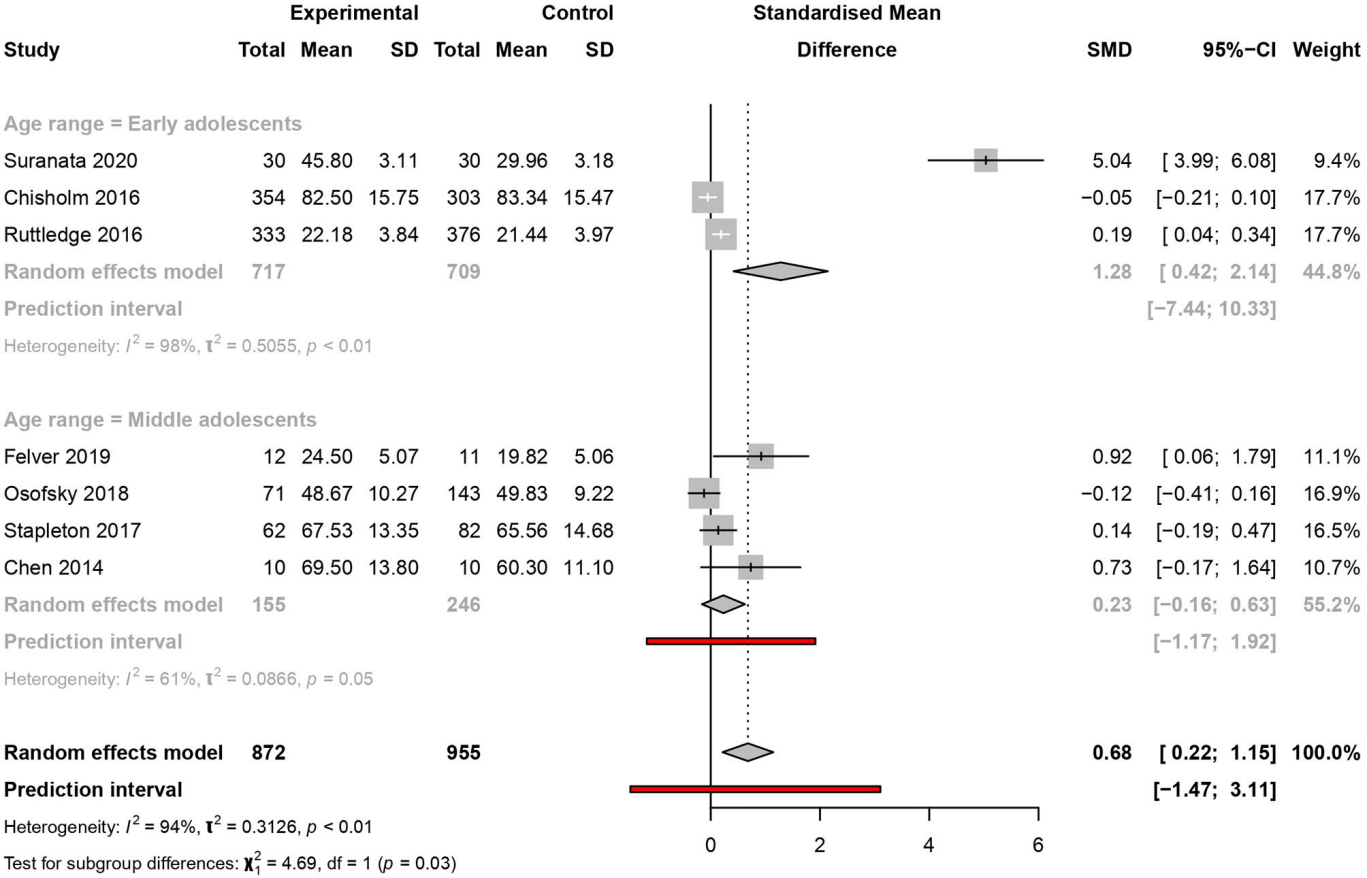
Meta-analysis of the effectiveness of school-based interventions for adolescents according to age range.

The meta-analysis showed statistical differences among studies when categorised as early adolescence (aged 10–13) [SMD = 1.28, 95% CI (0.42–2.14), PI (−7.44 to 10.33)] (*I*^2^ = 98%, *p* < 0.01) and middle adolescence (aged 14–17) [SMD = 0.23, 95% CI (−0.16 to 0.63), PI (−1.17 to 1.92)] (*I*^2^ = 61%, *p* = 0.05). No studies were identified for late adolescence (≥18 years of age) (Figure 6).

### Sensitivity analysis

#### Duration of the intervention

**Figure 7:**
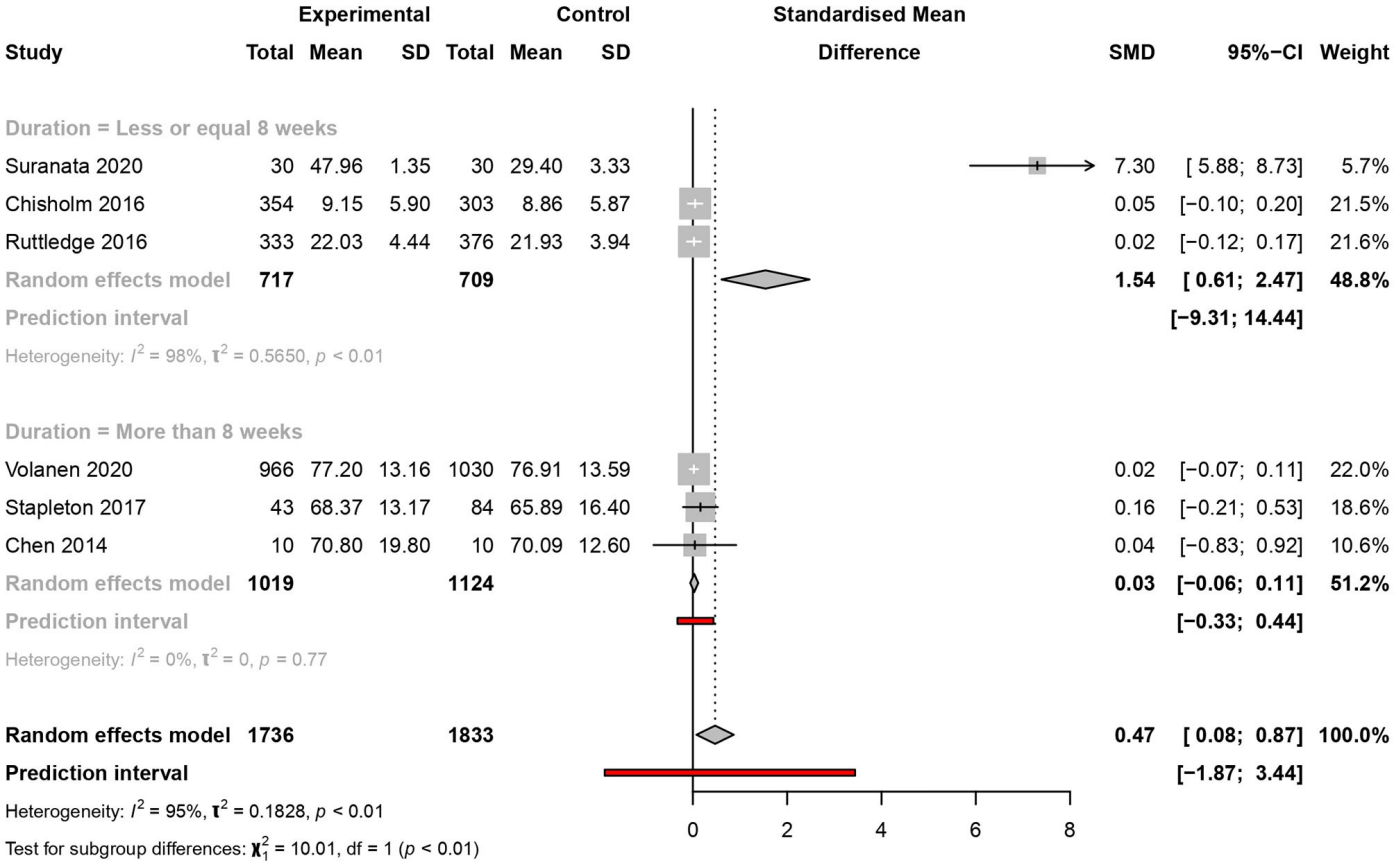
Meta-analysis of the effectiveness of school-based interventions for adolescents according to follow-up—sensitivity analysis.

There were six studies, three per subgroup, that reported the results of their follow-up of more than 8 or 8 weeks or less. Significant results of the intervention were obtained only in the subgroup with a follow-up of 8 weeks or less [SMD=1.54, 95% CI (0.61–2.47) and PI (−9.31 to 14.44)] with considerable heterogeneity (*I*^2^ = 98%, *p* < 0.01) but not for the subgroup with more than 8 weeks of follow-up (Figure 7).

#### Quality of the studies

The meta-analysis was performed according to each of the parameters of the Cochrane risk of bias tool, except for the blinding of participants and personnel, which, as discussed earlier, showed a high risk of bias for all the included studies due to the impossibility of masking any group, owing to the nature of the intervention. Those studies marked yellow or green for any of the Cochrane scale parameters that were considered for the meta-analysis. The studies with a high risk of bias (red) were excluded from the sensitivity analysis.

The analysis examined factors including the adequacy of the generation of the allocation sequence, the concealment of allocation to treatment conditions, blinding of outcome assessment, handling of incomplete data, selective outcome reporting, and other possible risks of bias. The results showed a statistically significant increase in resilience SMD ranging from 0.58 to 1.17 [95% CI (0.29–1.67)].

### Publication bias analysis

**Figure 8:**
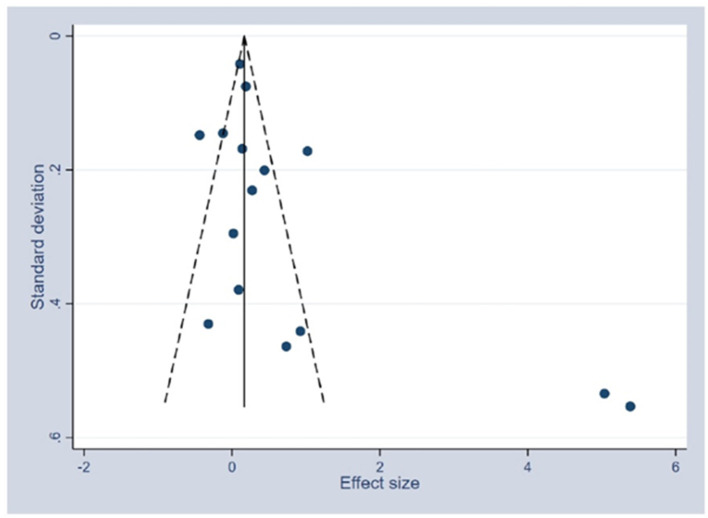
Funnel plot of the publication bias assessment—systematic review of school-based interventions for adolescents.

Taking into account the effect of magnitude and associated standard error, publication bias was evaluated visually using the funnel plot. The funnel plot did not show considerable asymmetry. Nevertheless, there were two outliers in the included studies (Figure 8). The results of the asymmetry Egger test showed no evidence of publication bias (*p* = 0.076).

## Discussion

Our systematic review with meta-analysis examined the type and effectiveness of interventions delivered in schools to increase resilience among adolescents in the general population and at risk. To our knowledge, this is the first systematic review with meta-analysis that has studied together the type of intervention and the target population of the intervention (at-risk or general population) to assess the effectiveness of resilience interventions in schools.

### Characteristics of the studies

A total of 27 articles on different resilience interventions were included in the systematic review. Our results showed that most of the techniques used to increase resilience were multicomponent (a combination of more than one technique), contrary to other reviews, where CBT was the most used intervention to increase resilience (Dray et al., 2017; Pinto et al., 2021).

Our study identified a spectrum of sessions ranging from 1 to 23, very similar to the study of Leppin et al. (2014), who reported interventions ranging from 1 to 24 sessions, or Pinto et al. (2021), who reported sessions that ranged from 5 to 23, and Dray et al. (2017), who reported interventions including a curriculum component that ranged from one lesson per week up to daily lessons during 5 to 32 weeks. The mean duration for the sessions was 59.33 min, ranging between 45 and 120 min. Our finding was in agreement with other reviews that ranged from 10 to 120 min (Pinto et al., 2021) and from 40 to 150 min (Leppin et al., 2014).

Although all programmes included in this review aimed to increase resilience and protective factors, the specific skills taught and the outcomes in each programme differed. This was similar to the study by Fenwick-Smith et al. (2018), and it may be explained by the variability and difficulty of defining resilience (Aburn et al., 2016) and creating programmes around the topic (Kaufman et al., 1994).

Most of the interventions found in the literature were focused on individual factors that fit with the Individual and Environmental Resilience Model (IERM) (Llistosella et al., 2022). Among them, we found that social-emotional competence, managing stress and anxiety, self-awareness, and coping skills were highlighted. Additionally, we found that the cognitive technique of problem-solving was one of the most used in resilience interventions.

Concerning the protective factors related to the environment, the majority of the interventions focused on social and school support (Slone et al., 2013; Gudiño et al., 2016; Scarf et al., 2017; Mirza and Arif, 2018; Kuperminc et al., 2020) and peer relationships (Sarkar et al., 2017; Kuperminc et al., 2020; Maalouf et al., 2020). Other factors were also identified using the IERM (Llistosella et al., 2022). In line with other reviews, 21 different scales were found to measure resilience; however, the SDQ and the CD-RISC (Dray et al., 2017; Pinto et al., 2021) scales were the most used ones.

### Effectiveness of interventions

The results from our meta-analysis revealed that certain types of resilience-based interventions were significantly beneficial. In particular, interventions using multicomponent and CBT increased resilience; the effect size was similar to other studies (Dray et al., 2017; Pinto et al., 2021). Interventions such as social and emotional learning, counselling or mentoring, mindfulness, and contact were not significant; it appeared that they did not increase resilience by themselves. This could be explained by the many protective factors related to resilience (Llistosella et al., 2022). Therefore, it would make sense to use multicomponent techniques that encompass several protective factors. In the case of counselling or mentoring, we found a large confidence interval, explained by the extreme values of the two included studies. Here, we hypothesise that it would be significant with the inclusion of more studies. In the case of contact intervention, it could also be explained by the lack of studies, as we only had one. However, interventions focused on self-awareness, such as mindfulness or yoga, did not show efficacy, similar to another study reported by Joyce et al. (2018). However, adolescents were not included in that study. Another review by Pinto et al. (2021) demonstrated the effectiveness of interventions in adolescents, including mindfulness, but it was not independently analysed.

Our subgroup analyses revealed that the effectiveness of the interventions only occurred in populations at risk and early adolescents. These results are in line with Fenwick-Smith et al. (2018); although resilience-promoting programmes in that study did not specifically target at-risk children, they did support positive change and growth, especially among children at risk. In general, previous systematic reviews (Dray et al., 2017; Pinto et al., 2021) do not stratify by general population or population at risk.

Our results also showed that interventions should be delivered as soon as possible, before middle adolescence. Interventions that are delivered during early adolescence would fix concepts and help acquire the skills that would be important for the development of resilience in the future. However, family, relational, behavioural, emotional, and environmental mediators, which may seem relevant for the effectiveness of these interventions, have not been meta-analysed due to the analytical limitations of identified studies. For example, a systematic review reported that adequate behavioural control of adolescents' peer behaviour and a more positive balance in their relationships with their parents seemed to buffer the effects of mental health problems, increasing their effectiveness. Unfortunately, other mediators, such as emotional, cognitive, and, more importantly, environmental, such as low neighbourhood socioeconomic status, delinquency, exposure to adverse events in the population at risk, or high rates of substance use, are strikingly neglected in the literature (Mestre et al., 2022).

When we analysed the effectiveness of interventions over time, our results showed that it was significantly effective up to 8 weeks but not beyond. In contrast, previous reviews showed that intervention effects were maintained for up to 3–12 months (Pinto et al., 2021). However, interventions in any setting, including online interventions and combined strategies for parents and children, were found to differ from exclusive school-setting interventions.

The predictive interval provides more uniform and accurate estimates of effects in a study, thus facilitating the generalisation of results to clinical practise or community settings. Our results showed that, although these interventions appeared effective in increasing resilience in our meta-analysis, these interventions might not be effective when applied to school settings. Specifically, some interventions delivered to at-risk populations over short durations (<8 weeks) and early adolescence that include multicomponent and CBT therapies may not be effective when applied.

The findings from our study could be useful for school or community nurses, educational psychologists, social workers, and other professionals who are involved in designing strategies for improving or reinforcing resilience and protective factors related to resilience in adolescents. Based on our findings, the following recommendations for school practise could be considered: (a) multicomponent interventions and CBT that focus on strengthening protective factors of at-risk adolescents can be effective in improving their resilience and should be considered in the implementation of resilience promotion programmes; (b) monitoring mechanisms that should be established to periodically measure the impact of the interventions; and (c) to achieve long-term effects, retain effectiveness, and sustainability of school interventions, it is recommended to offer short versions of the intervention to adolescents during the follow-up, include them as part of the school curriculum, and involve the school directors and teachers, and families.

### Limitations

This study had some limitations that should be considered. First, only 16 studies (59.2%) were included in the meta-analysis due to a lack of available data or a lack of a control group. Additionally, performing sensitivity analysis across different subgroups, such as early and middle adolescence, or type of intervention, was very limited by the number of included studies. Further, other potential mediation variables, as mentioned above, were assessed in this systematic review. For studies with incomplete data, the corresponding authors were not contacted. However, the robustness of our meta-analysis was assessed with the Cochrane risk of bias tool, and the sensitivity analysis was performed based on the quality of the studies. Most of the included studies were rated as having an overall or unclear high risk of bias in several domains, in agreement with other reviews of resilience (Pinto et al., 2021) and psychological programmes (Dray et al., 2017), which could affect the results.

Second, in the absence of a universal definition of resilience (Aburn et al., 2016), more than 60 different protective factors were found to be involved in the resilient process (Llistosella et al., 2022), which is why, although all interventions aimed at increasing resilience, the focus or enhanced protective factors were different. Consequently, qualitative and quantitative heterogeneity of the included studies was found in terms of design, type of interventions, follow-up, characteristics of the participants, and outcome assessment. Given the diversity of the studies and the protective factors involved in the resilience process, this expected heterogeneity was also found in previous systematic reviews (Dray et al., 2017; Pinto et al., 2021). Although most of the studies included a control group, allowing the comparison of results, only 13 studies had performed randomisation. These studies presented little evidence compared to those that used randomisation. In addition, three articles (Ijadi-Maghsoodi et al., 2017; McAllister et al., 2018; Sugiyama et al., 2020) did not include a control group to compare results, further weakening their evaluation. For example, Sugiyama et al. (2020) applied CBT to 229 students affected by heavy rains in Hiroshima, reducing depression and improving resilience. However, the absence of a control group makes it difficult to identify whether the increased resilience and the reduction in depression were because of their programme or simply a natural developmental progression (Dray et al., 2017). Further, the types of risk that the at-risk population was exposed to were very varied, ranging from low resilience to natural disasters. Despite this heterogeneity, the results were significant for this population group.

Third, only Spanish and English publications were considered, and studies from other languages were not included. Further, the search strategy was limited to the last 10 years. Some studies were included that reported data related to participants under 10 years, though they also included our target population; therefore, the findings of our review on interventions are not exclusive to adolescents aged 10–19 years.

Despite these limitations, this review and meta-analysis were conducted with high methodological rigour. Additionally, to our knowledge, this is the first review that analyses the effectiveness of resilience-based interventions delivered in schools among adolescents, by population subgroups, and by type of interventions.

## Conclusions

Findings from this study support the applicability and benefits of resilience-based interventions in schools. In particular, it supports the benefits of interventions using CBT as a core intervention along with other multicomponent interventions for increasing the effectiveness of resilience among at-risk adolescents, although some interventions may not be effective when applied. Our findings also indicate that the effectiveness lasts up to 8 weeks and among an early adolescent population. All the interventions examined in our systematic review aimed to increase resilience and protective factors, but specific skills taught and outcomes obtained in each intervention were different. Therefore, further research is needed to better identify the key elements or skills that increase resilience the most and also among adolescents from the general population. Further, the necessary elements or strategies to prolong the effect of the interventions need attention.

## Data availability statement

The original contributions presented in the study are included in the article/Supplementary material, further inquiries can be directed to the corresponding author.

## Author contributions

ML: conceptualisation, methodology, data extraction, interpretation of data, supervision, and writing—original draft preparation. BG-F and LM-D: conceptualisation, methodology, data extraction, and writing—reviewing. AM-M and PC: methodology, analysis and interpretation of data, and writing and reviewing. CP-V: methodology, data extraction, and writing and reviewing. BFM: analysis and interpretation of data. All authors have approved the submitted version.
