# Supplementary material for: Effectiveness of resilience-based interventions in schools for adolescents: a systematic review and meta-analysis
Source: Front Psychol. 2023 Oct 6;14:1211113. doi: 10.3389/fpsyg.2023.1211113 (PMC10587685; doi:10.3389/fpsyg.2023.1211113)
Supplement: Supplementary file 2 [file Table_2.DOCX]

**Supplementary table 2.** Search strategy for the systematic review of the effectiveness of resilience-based interventions in schools for adolescents

| Database | Strategy |
| --- | --- |
| MEDLINE/PUBMED | **((((((((("Parent-Child Relations"[Mesh]) OR "Interpersonal Relations"[Mesh]) OR "Social Participation"[Mesh]) OR "Students"[Mesh]) OR "Emotional Regulation"[Mesh]) OR "Empathy"[Mesh]) OR "Self Concept"[Mesh]) OR "Adaptation, Psychological"[Mesh] OR ("Social Support"[Mesh])) AND (("Resilience, Psychological"[Mesh] OR resilience) AND ("Psychosocial Intervention"[Mesh] OR intervention)) Filters: Adolescent: 13-18 years, from 2010 - 2021** |
| EMBASE | ('parent-child relations'/exp OR 'parent-child relations' OR 'interpersonal relations'/exp OR 'interpersonal relations' OR 'social participation'/exp OR 'social participation' OR 'students'/exp OR 'students' OR 'emotional regulation'/exp OR 'emotional regulation' OR 'empathy'/exp OR 'empathy' OR 'self concept'/exp OR 'self concept' OR 'adaptation, psychological'/exp OR 'adaptation, psychological' OR 'social support'/exp OR 'social support') AND ('resilience, psychological'/exp OR 'resilience, psychological' OR 'resilience'/exp OR resilience) AND ('psychosocial intervention'/exp OR 'psychosocial intervention' OR 'intervention'/exp OR intervention) Filters: 2010-2021 |
| Web of Science | TS=(("Parent-Child Relations"[Mesh] OR "Interpersonal Relations"[Mesh] OR "Social Participation"[Mesh] OR "Students"[Mesh] OR "Emotional Regulation"[Mesh] OR "Empathy"[Mesh] OR "Self Concept"[Mesh] OR "Adaptation, Psychological"[Mesh] OR "Social Support"[Mesh]) AND ("Resilience, Psychological"[Mesh] OR resilience) AND ("Psychosocial Intervention"[Mesh] OR intervention) AND (Adolescent)) and 2021 or 2020 or 2019 or 2018 or 2017 or 2016 or 2015 or 2014 or 2013 or 2012 or 2011 or 2010 (Publication Years) |
| PsycInfo | (parent-child relations OR interpersonal relations OR social participation OR students OR emotional regulation OR empathy OR self concept OR adaptation, psychological OR social support) AND (resilience psychological OR resilience) AND ( psychosocial intervention OR intervention): **Adolescent: 13-17 years, from 2010 - 2021** |
